# Epidemiological analysis of cardiac trauma victims at a referral trauma hospital: a 5 year case series

**DOI:** 10.1590/0100-6991e-20223120

**Published:** 2022-02-18

**Authors:** LUCAS MANSANO SARQUIS, ARNON CÉSAR BRUNET-SCHULTZE, BRUNO BERARDI GAZOLA, IWAN AUGUSTO COLLAÇO, ALAN JUNIOR DE AGUIAR, HECTOR FONTES

**Affiliations:** 1 - Hospital do Trabalhador, General Surgery - Curitiba - PR - Brasil; 2 - Faculdades Pequeno Príncipe, Faculty of Medicine - Curitiba - PR - Brasil

**Keywords:** Heart, General Surgery, Myocardial Contusions, Coração, Traumatismos Cardíacos, Cirurgia Geral

## Abstract

**Objective:**

to describe, analyze, and trace the epidemiological profile for cardiac trauma victims on a referral trauma hospital of a major urban center.

**Methods:**

a case series study to review, describe, compile and analyze medical records of all patients sustaining traumatic cardiac injuries, from January 2015 to January 2020 admitted to the referral trauma hospital of Curitiba, Brazil. Patients sustaining traumatic heart injuries were identified using the hospitals database. Patients who died prior to reaching hospital care were excluded.

**Results:**

all 22 cases were urban victims, mostly penetrating injuries (12 stab wounds, 9 gunshot wounds); 82% were male; mean age, 37.1 years. 17 cases (77%) occurred during night hours, 15 between Friday and Sunday, and 15 were admitted hemodynamically stable. Only 27% were diagnosed with FAST, the remainder requiring other imaging exams. About incisions, 14 had thoracotomies, 6 median sternotomies and in 2 cases both. Of injuries, 8 affected the right ventricle, 3 right atrium, 9 left ventricle, 1 right coronary sulcus and 1 anterior wall. All had cardiorrhaphy repair. 3 patients died, 17 were discharged and 2 were transferred. 17 received postoperative echocardiograms, revealing ejection fractions ranging 55.1% to 75%. Patients spent a mean of 9.6 days on ICU and a mean of 15.2 days of total hospital stay. The mortality rate was 14%.

**Conclusions:**

cardiac traumas predominantly occurred in adult males, due to violent causes, during night hours on weekends. The overall mortality rate found (14%), as well as total hospital stay, accords with the literature.

## RESUMO

**Objetivo:**

descrever, analisar e traçar o perfil epidemiológico das vítimas de trauma cardíaco em hospital de referência em trauma de grande centro urbano.

**Métodos:**

uma série de casos para descrever e analisar prontuários de todos os pacientes que sofreram lesões cardíacas traumáticas, entre janeiro, 2015, a janeiro, 2020, admitidos no hospital referência em trauma de Curitiba, Brasil. Pacientes que sofreram lesões cardíacas traumáticas foram identificados no banco de dados do hospital. Pacientes em óbito antes da chegada aos cuidados hospitalares foram excluídos.

**Resultados:**

todos os 22 casos foram vítimas urbanas, maioria ferimentos penetrantes (12 por arma branca, 9 por arma de fogo); 82% homens; idade média 37.1 anos. 17 casos (77%) ocorreram no período noturno, 15 entre sexta-feira e domingo. 15 foram admitidos hemodinamicamente estáveis. 27% diagnosticados com FAST; demais demandaram outros exames. Das incisões, 14 receberam toracotomias, 6 esternotomias medianas, 2 casos ambas. Das lesões, 8 afetaram ventrículo direito, 3 átrio direito, 9 ventrículo esquerdo, 1 sulco coronário direito, 1 parede anterior. Todos receberam cardiorrafias. 3 pacientes morreram, 17 tiveram alta e 2 foram transferidos. 17 receberam ecocardiograma pós-operatório, revelando frações de ejeção de 55.1% a 75%. Os pacientes passaram em média 9.6 dias em UTI e 15.2 dias de internamento hospitalar total. A taxa de mortalidade foi de 14%.

**Conclusões:**

traumas cardíacos ocorreram predominantemente em homens adultos, devido a causas violentas, durante o período noturno nos finais de semana. A taxa de mortalidade encontrada, assim como o tempo total de internamento hospitalar, esteve em acordo com a literatura.

## INTRODUCTION

Cardiac injuries are among the most severe and lethal conditions that can occur in trauma victims, being surpassed only by central nervous system injuries as the leading cause of death^1^. Some of the most severe available reports have even showed mortality rates that can reach up to 95% at the pre-hospital environment following with up to 50% of the victims that survive to reach the hospital in some cases^1^ ^,^ ^2^.

Cardiac traumas are classified primarily by their mechanism of injury, which may be penetrating or non-penetrating (blunt cardiac injury). Blunt cardiac injury is reported as being the most common, being majorly caused by abrupt deceleration resulting from car accidents (50%), followed by pedestrian hit-and-run (35%), motorcycle accidents (9%) and falls from height (6%)^1^ ^,^ ^3^, while penetrating cardiac injuries are mostly due to stab wounds (59%), gunshot-injuries (26%) and other causes (15%)^4^.

The initial hemodynamic state presentation is a major factor for the outcome of these cases, being clearly evidenced by the poor survival rates of the instances in which patients are admitted to emergency departments already in shock, which are of 35% after penetrating injuries and a mere 2% after blunt cardiac traumas^5^.

Another major factor for the outcome is the diagnostic management. Hemodynamically stable patients may be screened for cardiac trauma with electrocardiography, with a sensitivity of 89%, or computed tomography, with sensitivity of up to 100% and specificity of 96% for hemopericardium^2,^ ^3^. These exams are preferred instead of troponin dosing, since the latter showed a sensitivity of only 23%^2^. Nevertheless, the most important tool for rapid assessment of cardiac trauma injuries in the emergency department, especially for hemodynamically unstable cases, is the FAST (Focused Assessment with sonography in trauma) exam^4^.

In addition to the variables in diagnosis, the different surgical techniques that can be employed for repair are also reported to play a major role on the outcome, although median sternotomy cardiorrhaphy is the most common approach. Special attention should also be given to the different types of injuries that can be found, noting however, that in most cases those are found on the right ventricle^2^ ^,^ ^5^ ^,^ ^6^.

### Objective

Considering the high severity and number of relevant variables that impact the outcome of cardiac trauma injuries, it is therefore necessary to describe and analyze the data from previous cardiac trauma cases on referral trauma hospitals to further the current scientific evidence and improve the basis for future intervention initiatives aiming to optimize the care for these critical patients. This research aimed to describe the data from previous cardiac trauma cases at the referral trauma hospital of a large urban center, and to analyze them based on current evidence, seeking to trace the epidemiological profile of the victims of this condition at that setting.

## METHOD

 This study was conducted with the approval of the Referral Hospital’s Research Ethics Committee, on June 25, 2020, under the CAAE (Ethics Appreciation Presenting Certificate): 33118320.0.0000.5225. Considering that no direct contact was established with the selected patients, limiting the study to the usage of previously recorded documents (medical records, test results, archived images, etc), and also considering that it would be unfeasible to directly contact all patients (unknown address/telephone number, deaths), the Ethics Committee waived the requirement for a free and informed consent form. Nonetheless, all authors assure their rigorous commitment in preserving the confidentiality of all patient’s private information included in the study and have done so by taking several necessary precautions to ensure respect and anonymity to those involved, which most importantly included, but were not limited to, refraining from reporting identity compromising data (individual name and surname, neighborhood of residence, exact date and hour of occurrence).

### Patient Selection

The selected hospital is the referral for severe trauma in Curitiba, Brazil, a state capital city, with an estimated population of 1.9 million during this period. Patients sustaining traumatic heart injuries were identified using the hospital’s database, therefore, patients who did not survive to reach hospital care were excluded from the study. All patients that sustained traumatic heart injuries and underwent treatment at the referral trauma hospital during the 5 year period of January 2015 to January 2020, were included.

#### Study Design and Data Collection

A case series descriptive study was conducted through the compilation and analysis of the relevant data and epidemiological variables of electronic medical records and test results of the selected patients. The variables assessed were: sex; age; neighborhood of residence; date, day of the week and hour of occurrence (cases that occurred between 06:00 PM and 05:59 AM were considered night hour occurrences); mechanism of trauma and if the reported mechanism had a violence related onset (stab wound, gunshot injury, physical assault) or non-violence related onset (motor vehicle collision, accidents); whether there was associated cranioencephalic trauma; if imaging tests were required for diagnosis, and if so, which, what their report was and whether they were performed intraoperatively or not; first admission laboratory test results (Hb, MCV, pH, PCO2, HCO3, base excess); requirement of transfusion; admission vital signs (HR, BP, RR); admission Glasgow score; whether there were signs of drug use; comorbidities; whether the initial management was surgical or conservative; type of incision used; pattern, location and size of the injury found; presence of associated injuries; treatment approach (type and size of suture thread); number and size of immediate postoperative drains; if pericardial drainage was required; if there were surgical complications; if specifically pneumonia or wall infection occurred; whether intensive care was required and if so, for how many days; total length of hospitalization; if bacteremia occurred; blood culture results; number of days under orotracheal intubation in the postoperative period and, if so, for how long was the patient kept on mechanical ventilation; if tracheostomy was required; outcome of the hospitalization (discharge, interhospital transfer, death or discharge against medical advice); and, finally, outpatient outcome.

## RESULTS

A total of 22 patients presented with traumatic heart injuries at the selected trauma referral hospital in the 5 year period analyzed. After reviewing electronic medical records, no cases were excluded. A final sample of 22 traumatic heart injury cases comprised the study group.

Data pertaining the most relevant findings are shown in Table 1. All 22 cases were urban victims due to violent mechanisms, two of them in 2015, seven in 2016, five in 2017, five in 2018 and three in 2019. The penetrating mechanism accounted for the majority of injuries (95%), being twelve of those by stab wounds, nine by gunshot wounds and only one patient, who was found unconscious in a public road, suffered blunt cardiac trauma, as a result of physical assault. Ten of the twenty-one victims of penetrating mechanism had the cardiac injury as an isolated injury, while the rest also presented with associated injuries.

**Table 1:** Data relation of the most relevant epidemiological, diagnostic, treatment, surgical, and outcome variables.

Variables	Total (n=22)	Percentage of total cases
Sex (male)	18	82
Patient age group (y)		
11-20	1	5
21-30	3	14
31-40	11	50
41-50	6	27
51-60	1	5
Mechanism of trauma		
Stab injury	12	55
Gunshot injury	9	41
Blunt injury	1	5
Weekday period of occurrence		
Monday to Thursday	7	32
Friday to Sunday	15	68
Imaging exams		
FAST	6	27
CT	16	73
X-ray	5	23
Surgical access		
Only left anterolateral thoracotomy	9	41
Only median sternotomy	6	27
Additional incisions	7	32
Variables	Total (n=22)	Percentage of total cases
Cardiac injuries		
Right Ventricle	8	36
Right Atrium	3	14
Left Ventricle	9	41
Anterior Wall and Apex	1	5
Right Coronary Sulcus	1	5
Surgical conduct		
Cardiorrhaphy	22	100
Required blood transfusion	14	64
CPR	3	14
Associated injuries		
Abdominal and Diaphragm	4	26.6
Cranioencephalic trauma	1	6.6
Lungs and thoracic arteries	5	33.3
Hemo/Pneumothorax	2	13.3
Spinal cord	1	6.6
Rib Fracture	2	13.3
Post-surgical outcome		
Death	3	14
Discharge	17	77
Interhospital transfer	2	9
Echocardiograms with EF >55%	17	77

Values are presented as number of cases and corresponding percentage of total cases (%). FAST, Focused Assessment with Sonography in Trauma; CT, Computed Tomography; CPR, Cardiopulmonary Resuscitation; EF, Ejection Fraction.

Most of the victims were men (82%), with an age range of 19 to 55 years, mean age of 37.1 years. Women’s mean age (32.5 years) was lower than that of men (37.1 years). Fifteen of the twenty-two cases occurred between Friday and Sunday. Most of the cases were also admitted in night hours (77%).

At admission, fifteen patients were hemodynamically stable and seven hemodynamically unstable. During investigation, the FAST was the only imaging exam required for definitive diagnosis of cardiac injury in only one case, although it revealed pericardial fluid in the pericardial window in four other patients. CT (Computed tomography) was necessary for diagnosis in sixteen cases, seven of whom were of the angiotomography type, eight chest scans and one full-body scan tomography (skull, cervical, chest, abdomen and pelvis) for the exceptional case of the patient found unconscious in a public road, who also had, along with an uncertain history, evidence of cranioencephalic trauma and an admission Glasgow score of 6. Computed tomography was the only imaging exam performed in five of the sixteen cases in which it was requested. Finally, chest X-rays were performed for five patients, and in two of these cases, it was the only imaging exam.

All 22 traumatic heart injury cases were treated by cardiorrhaphy, meaning conservative management was not performed in any instance in the selected period.

Of the used incisions, left anterolateral thoracotomy was chosen in nine cases, median sternotomy in six, and seven additional incisions: five bilateral thoracotomies, and two left anterolateral thoracotomies combined with median sternotomies.

Cardiac lesions found during operation, were predominantly in the left ventricle, being present in 41% of the cases, while there were 36% right ventricle lesions. There were also three lesions in the right atrium, one in the right coronary sulcus and one in the anterior wall and apex. 

Associated lesions were described in thirteen of the twenty-two patients, being two of them rib fractures, one spinal cord injury, one left mammary artery injury, three abdominal injuries, one diaphragmatic injury, one traumatic brain injury (subarachnoid hemorrhage), four pulmonary lobe injuries, one pneumothorax and one hemopneumothorax. Blood transfusions were required by 14 (63%) of the patients, with a median of 4 U (1-12 U) of packed red blood cells as part of the resuscitation efforts.

There were three cardiac arrests during the operation of three different patients, with two of them returning to normal sinus rhythm after open resuscitation under direct cardiac compressions and aortic clamping, for 18 and 20 minutes, respectively. The third case underwent the same procedure but was declared dead after 25 minutes.

Regarding hospital outcome, seventeen patients were discharged, two were transferred to other hospitals and three died. Only these two transferred cases resulted in a loss of follow up, which translates into a rate of 4.4% for this study. Of the three deaths, one occurred during the operation and two in the postoperative period, respectively on the first and tenth day of hospitalization. The two patients that died on postoperative period, had five or more penetrating injuries, meaning they sustained other injuries in addition to the cardiac trauma. The patient who died during the operation had multiple associated lesions, including diaphragmatic injury, posterior stomach lesion, small bowel injury, liver injury and a reported presence of hematoma in the retroperitoneum. All patients who died were admitted in the hospital hemodynamically unstable.

Postoperative echocardiogram was performed in seventeen patients, and it showed values ranging between 55.1% and 75% (mean of 66.2%). Nine of them had evidence of mild effusion in the pericardium, with no signs of tamponade or restriction of cardiac relaxation. Only one revealed a large volume effusion and signs of cardiac tamponade.

Regarding postoperative complications, one case of pleural empyema was reported. This patient had a hemopneumothorax at admission and died on the tenth day of hospitalization due to sepsis. A total of 21 patients required post-operative ICU (intensive care unit) and hospital care, with the only exception being the patient who died during operation, as mentioned. Excluding this case for that reason, the mean length of ICU stay was 9.6 days, and the mean length of total hospital stay was 15.2 days.

## DISCUSSION

Trauma due to violent causes among the young population represents a significant portion of the demand of emergency surgical services, especially in large urban centers. Cardiac trauma can be classified as blunt or penetrating^1^, and according to current evidence, its mortality rate considering only the cases who don’t survive to reach the hospital ranges from 80% to 95%^1^ ^,^ ^2^ ^,^ ^4^ ^,^ ^5^. For the patients who do reach the hospital, it has been shown a predominance of this type of trauma in the young male population with a mean age of 30.8 years^7^, which roughly matches the data we found in our study, since 82% of the cases were men, although with a fairly higher mean age of 37.1 years.

To our knowledge none of the literature studies specifically report data with regards to cardiac trauma injury and the variables of specific hours of the day or weekdays as a potential association between them, therefore, no comparisons can be made. In our report, there was a higher occurrence of cardiac trauma injuries occurring during night hours (77%) and during weekends (68%), suggesting that these findings are relatively novel.

The most frequent blunt injury mechanism in developed countries are abrupt decelerations, such as those that occur in vehicle collisions or in sporting events^3^ ^,^ ^5^. Penetrating cardiac injuries come with a mortality rate of approximately 40%^2^, mostly by stab wounds, which is also the most frequent mechanism in developing countries^5^, but also frequently by gunshot wounds, which are often more lethal than the former^8^. Since this research was conducted in Brazil, a developing country, our study has shown evidence that supports those statements. Regardless of the mechanism, however, it was expected for the right ventricle to be the most frequently injured, based on the literature, which stated that it occurred in 53% of the cases, followed by 32% of involvement of the left ventricle^5^. It is noteworthy that our study showed they had significantly similar rates of injury, with even a higher incidence for the left ventricle (41%), as opposed to only 36% of the cases showing right ventricle injuries.

Among the most common associated injuries, a study involving 1,359 patients with thoracic trauma showed 49% of them had rib fractures, 20% had pneumothorax, 12% had pulmonary contusion and 6% had thoracic vascular injury^9^. When comparing those numbers with our findings, it can be said they were disparaging at best. Most notably, our study showed a high incidence of abdominal and diaphragm injury, which is barely of any note in the former report, and on the other hand, while it showed that rib fractures play a major role, in our case they only accounted for 13.3% of the associated injuries. Nevertheless, it must also be noted that that study also showed thoracic vascular injury with an importance quite close to our findings of 6.6%.

Pre-hospital care needs to be fast, being essential to ensure initial stabilization and transportation to the trauma center for definitive treatment^10^. The major factors that translate into an increased mortality rate include total scene and transit time longer than ten minutes, the need for CPR (cardiopulmonary resuscitation), exsanguination, decreased Glasgow Coma Scale, massive hemothorax, need for resuscitative thoracotomy, hypotension (Systolic BP <75mmHg) and bradycardia (HR <50bpm)^11^. In the modern era, the ability to save the lives of patients suffering from penetrating cardiac injury is an important marker of quality of a trauma center^9^, and to achieve that, it requires proper diagnostic tools and a team qualified to manage the cases that survive to reach the hospital.

Of the diagnostic equipment, FAST has become widely available in the last decade^12^, bringing along its high sensitivity (92-100%) and specificity (99-100%) for pericardial effusions^4^, which in turn allows for an early identification (mean of 0.8 minutes)^13^ of the presence of free fluid around the heart and lungs, as well as in the abdominal cavity^3^. That said, it should be reminded that its accuracy still depends on the operator^12^, and as such, some authors consider complementing FAST with a transthoracic echocardiogram. The latter might show focal myocardial contusions or movement abnormalities, as well as secondary injuries such as ventricular septal defects, thrombus, or cardiac aneurysms, while also further decreasing the risks of a false negative FAST^3^. Nevertheless, the results of our study showed an unexpectedly lower use of this tool for diagnosing cardiac injuries, being performed on a mere 27% of the cases. Although FAST findings continue to be overridden by overall clinical assessment as the basis for final management decision^12^, there is still some divergence on whose patients should undergo the exam. For hemodynamically unstable cases, some authors advocate immediate operative exploration, restricting their use of FAST for stable cases, with the ones yielding a positive result being followed by a subxiphoid pericardial window (SPW) or other tests for confirmation before proceeding to the definitive treatment^7^. In contrast, other authors discourage the use of SPW due to concerns about possible uncontrolled hemorrhages, preferring instead to employ FAST extensively, for both stable and unstable patients, indicating surgical management for the unstable when a positive FAST is identified, and reserving SPW for when it is negative when clinical suspicion remains high^12^.

Another diagnostic tool widely used for hemodynamically stable patients^1^ is computed tomography, since it has 100% sensitivity and 96% specificity, for the diagnosis of hemopericardium^3^. It also allows surgeons to identify the trajectory of the injury and the structures affected^2^, a feature not achieved by the FAST. Our study has shown that CT scans were the most frequently used resource for diagnosis, being performed in 73% of the patients.

Chest X-rays could help identify a pneumothorax, a hemothorax, an increased cardiac area and bone fractures, as well as locating projectiles and ballistic fragments^14^, though in our hospital, it was required for only 23% of the cases.

The ECG has a reported sensitivity of 89%^2^ and could be considered an important component in the screening of hemodynamically stable patients, often showing sinus taquicardia^3^. Troponin dosage could also be performed, with a sensitivity of 23%^2^. Neither of those methods were used in any of the patients in the current study, which can be safely justified by the longer time they require to be performed or yield results, in comparison to the imaging exams.

The management of patients with cardiac trauma is usually surgical, rather than conservative, initially applying the concept of damage control, which involves the rapid containment of hemorrhage through cardiorrhaphy, followed by resuscitation and planned reoperation^2^. In this regard, the findings of our study showed the surgical approach being chosen as a rule, meaning none of the 22 cases were treated with a conservative management.

Generally, for hemodynamically unstable patients, the chosen surgical incision is the left anterolateral thoracotomy and for stable patients, median sternotomy^14^. Other options include the “claw” incision (bilateral anterolateral thoracotomy in the 5^th^ intercostal space plus a transverse sternotomy) and the extended anterolateral thoracotomy with transection of the sternum^14^. In our study, we found that at admission, fifteen patients presented hemodynamically stable, while the other seven were unstable. Although as mentioned, the choice of incision is not a rule, it was noticeable that our results deviated a bit regarding that, since despite having fifteen stable patients, only nine underwent left anterolateral thoracotomy as the only incision and six the median sternotomy as a sole incision. The remaining seven patients underwent either both of those incision options or one of the additional options.

The expected mortality rate based on the other studies was between 9.4%^10^ and 30%^13^, and the mean length of total hospital stay between 5 and 12.8 days^5^ ^,^ ^6^. Analyzing our findings, it can be said that these expectations were met regarding mortality rate, which was 14%, and though the mean length of total hospital stay was higher (15.2 days), the median of 11 days (range 1-75) was within expected. Interestingly, when those variables were analyzed separating cases in 2 groups, an association between blood transfusions and decreased hospital stay was seen, although without statistic comparisons, this cannot fully be supported. Patients who received blood transfusions spent less time in the hospital, with a mean length of total hospital stay of 10.2 days (Median 10, range 1-19), in contrast with those who did not receive blood transfusion, with a mean of 25.2 days (Median 22, range 6-75). Although a more focused study on this specific finding would be required for confirmation, it already suggests that blood transfusions may have a major impact on this outcome.

A study of the echocardiograms of patients undergoing cardiorrhaphy, performed before their discharge from the ICU, indicated that only 12% presented with a persistence or onset of new symptoms and abnormal findings. The others were asymptomatic and with a normal result, suggesting that the benefits of performing echocardiographic studies would be mostly reserved for patients who actually present with new symptoms or an abnormal exam after the surgical procedure^13^. The findings of our study, however, might suggest the opposite, since it was noted that under the same conditions, our postoperative echocardiograms, performed in seventeen of the twenty-two patients, revealed abnormal findings in 45%. Although this report was not a specific aim of this research, finding this contradiction was an unexpected, but noteworthy result.

### Study Limitations

The relatively small number of cases should be seen with caution, since that hinders statistical comparisons. Nonetheless, it should also be pointed out that even in large urban center hospitals, which are regional trauma referrals, the annual incidence of cardiac traumatic injury is low^1^ ^,^ ^5^ ^,^ ^6^. Furthermore, it is also important to mention that some of the medical records did not clearly contain all the variables initially proposed to be collected, though not an impediment to the reported findings, being the reason why the authors do not feel it changed the results in any significant manner.

## CONCLUSION

Low annual incidence of cardiac lesions occurred, predominantly in 30 to 50-year-old men, due to violent causes, mostly during night hours on weekends. These patients arrived at the hospital mostly hemodynamically stable, although the majority required blood transfusions. In addition, diagnostic management by imaging and surgical incision may vary depending on the case, while the surgical treatment approach is likely to be chosen as a rule. A slightly higher incidence of left ventricle injuries was seen, along with associated injuries and intraoperative complications. The overall mortality rate was similar or better than found in the literature.
